# The role of ^99m^Tc-MIBI SPECT/CT in patients with secondary hyperparathyroidism: comparison with ^99m^Tc-MIBI planar scintigraphy and ultrasonography

**DOI:** 10.1186/s12880-020-00517-9

**Published:** 2020-10-15

**Authors:** Shu-Qin Jiang, Ting Yang, Qiong Zou, Lei Xu, Ting Ye, Yin-Qian Kang, Wan-Ru Li, Ju Jiao, Yong Zhang

**Affiliations:** Department of Nuclear Medicine, The Third Affiliated Hospital of Sun Yat-Sen University, 600 Tianhe Road, Tianhe Street, Guangzhou, 510630 Guangdong China

**Keywords:** ^99m^tc-MIBI SPECT/CT, Secondary hyperparathyroidism, Sensitivity

## Abstract

**Background:**

This study aimed to compare the sensitivity of ^99m^Tc-MIBI SPECT/CT, ^99m^Tc-MIBI planar scintigraphy and ultrasonography (US) in patients with secondary hyperparathyroidism (SHPT), and to explore the factors that affect the sensitivity of ^99m^Tc-MIBI SPECT/CT.

**Methods:**

In this retrospective study, forty-six patients with SHPT who underwent ^99m^Tc-MIBI planar scintigraphy, ^99m^Tc-MIBI SPECT/CT and US were enrolled. They underwent surgery within 1 month. We compared the sensitivity of the different imaging methods based on the lesions according to the pathological results. The parathyroid lesions on ^99m^Tc-MIBI SPECT/CT images were divided into missed diagnosis group (MDG) and non-missed diagnosis group (NMDG). We compared the lesion to background ratio (LBR), maximum diameter, volume, the mean CT Hounsfield unit values (CT_mean_) and location of lesions between MDG and NMDG.

**Results:**

The sensitivity of ^99m^Tc-MIBI SPECT/CT, ^99m^Tc-MIBI planar scintigraphy and US were 70.30% versus 48.48% versus 61.82%, respectively. The sensitivity of ^99m^Tc-MIBI SPECT/CT combined US was 79.39%, which was higher than ^99m^Tc-MIBI SPECT/CT with significant difference (*P* = 0.000). On ^99m^Tc-MIBI SPECT/CT images, the LBR, maximum diameter and volume of lesions in MDG was smaller than those in NMDG with significant difference (*P* < 0.001). The average LBR, maximum diameter and volume of lesions in MDG and NMDG were 3.42 ± 1.28, 9.32 ± 2.69 mm, 208.51 ± 163.22 mm^3^ versus 6.75 ± 5.08, 15.03 ± 4.94 mm and 863.85 ± 1216.0 mm^3^, respectively.

**Conclusions:**

^99m^Tc-MIBI SPECT/CT exhibited the highest sensitivity among the three methods. When ^99m^Tc-MIBI SPECT/CT combined with US, the sensitivity can be further improved. Lesions with lower MIBI uptake and smaller lesions on ^99m^Tc-MIBI SPECT/CT images were easily missed.

## Background

Secondary hyperparathyroidism (SHPT) is a common and serious complication of chronic kidney disease (CKD), particularly among dialysis patients [1–3]. Long-term stimulation results in increased parathyroid hormone (PTH) secretion and parathyroid hyperplasia or even adenoma [4]. When patients do not improve with medical therapy, parathyroidectomy is recommended [5].

Currently, the most widely used methods in identifying abnormal parathyroid glands before surgery are ^99m^Tc-MIBI scintigraphy and ultrasonography (US) [6]. The procedures of ^99m^Tc-MIBI scintigraphy includes planar scintigraphy, SPECT and SPECT/CT. Most studies compared the sensitivitiy of ^99m^Tc-MIBI scintigraphy and US in primary hyperparathyroidism [7–10]. However, few studies compared the sensitivity of ^99m^Tc-MIBI scintigraphy and US in SHPT [11, 12]. Besides, the value of combined ^99m^Tc-MIBI scintigraphy and US in SHPT remains to be explored.

Since ^99m^Tc-MIBI SPECT/CT shows advantages in preoperative localization and anatomical depiction, its application is currently increasing in popularity [14, 15]. On ^99m^Tc-MIBI SPECT/CT images, the size, accurate location, density and adjacent tissues of the lesions can be observed. Several previous studies have mentioned that ^99m^Tc-MIBI SPECT/CT has a relatively low sensitivity in patients with multiglandular disease [11, 16], which usually occurs in SHPT. This means that some lesions may be missed during the imaging diagnosis. Several previous studies have mentioned some possible causes of missed diagnosis [11, 13, 17, 18], such as low uptake and small size of lesions.

Scine June 2016, ^99m^Tc-MIBI SPECT/CT combined with planar scintigraphy has been applied in our hospital. The primary aims of our study were to compare the sensitivity of ^99m^Tc-MIBI SPECT/CT, ^99m^Tc-MIBI planar scintigraphy and US in patients with SHPT, and to explore the sensitivity of ^99m^Tc-MIBI SPECT/CT combined with ^99m^Tc-MIBI planar scintigraphy or US. The secondary objectives were to compare the size, MIBI uptake and more other parameters between missed parathyroid lesions and non-missed parathyroid lesions on ^99m^Tc-MIBI SPECT/CT images.

## Methods

### Patients

In this retrospective study, 62 patients with SHPT caused by CKD who underwent ^99m^Tc-MIBI planar scintigraphy, ^99m^Tc-MIBI SPECT/CT and US from June 2016 to June 2019 in the Third Affiliated Hospital, Sun Yat-sen University were reviewd. Only 50 patients underwent parathyroidectomy and pathological examination within 1 month after imaging examination were futher evaluated. The exclusion criteria were: (1) patients with incomplete clinical data; (2) patients with Thyroid carcinoma. Finally, 46 patients were included in this study.

The clinical information (age, sex, years of CKD and dialysis, with or without thyroid disease), latest laboratory results (serum calcium, phosphorus, PTH, alkaline phosphatase) before surgery, imaging findings and pathological results of each patients were recorded. Patients with greater than or equal to one missed lesion were classified as the missed diagnosis group (MDG), and those without missed lesions were classified as the non-missed diagnosis group (NMDG).

The study was approved for retrospective analysis by the institutional ethics committee and the informed consent was waived.

### ^99m^Tc-MIBI scintigraphy

^99m^Tc-MIBI scintigraphy was performed using a SPECT/CT scanner (Symbia T6, Siemens, Germany) after an intravenous injection of 740 MBq ^99m^Tc-MIBI. Early and late planar scans of the neck and mediastinum were acquired at 15 min and 120 min, respectively. The planar images were recorded in a 128 × 128 matrix with a 20% window at a 140 keV photopeak using a low-energy, high-resolution parallel collimator. An additional SPECT/CT acquisition was performed immediately after the late planar scan. The SPECT acquisition comprised 32 views of 20 s each. CT acquisition was performed with a slice thickness of 2.5 mm, a current of 240 mAs, and a voltage of 110 kV. The images were reconstructed in the transverse, coronal, and sagittal projections.

### US

US was performed using a 4 to 15 MHz linear-array probe (Logiq7, GE, USA). The patient was in a supine position with the neck fully exposed, and the scan was performed from the upper to bilateral mandibular angle, lower to bilateral clavicle and bilateral to internal jugular vein. When abnormal parathyroid glands were observed, the number, location, size, shape, internal echo and blood supply were observed and recorded.

### Surgery and pathology

The choice of operation procedures was mainly based on the imaging results. If both US and nuclear medical examination suggested a single parathyroid lesion, minimally invasive parathyroidectomy was performed. If one of imaging results suggested that there were two or more parathyroid lesions, bilateral neck exploration was performed. During bilateral neck exploration, all the parathyroids explored were resected and one of them was cut into pieces and buried in the subcutaneous tissue of the forearm.

Intraoperative frozen-section examination was performed to confirm whether the resected tissue was parathyroid. After operation, pathological examination performed in all resected tissues. The size, color, texture and pathological classification of all resected tisues were reported.

### Imaging analysis

Two experienced nuclear medicine physicians evaluated the images of planar scintigraphy, SPECT/CT and US together, and a final consensus was reached. To explore the interobserver variability of ^99m^Tc-MIBI SPECT/CT, two other physicians evaluated the SPECT/CT images. Both observers were blinded to the pathological results. ^99m^Tc-MIBI SPECT/CT and ^99m^Tc-MIBI planar scintigraphy were evaluated independently at different days. ^99m^Tc-MIBI SPECT/CT was considered to be positive if a nodule on CT images with focal activity on SPECT images was detected. ^99m^Tc-MIBI planar scintigraphy was considered to be positive if the focal activity was detected on both early and late planar images. US was considered to be positive if well-defined, spherical or elliptical, hypoechoic or anechoic lesion was detected.

On SPECT/CT images, lesions with negative diagnoses and positive pathologies were classified as the MDG, and those with positive diagnoses and positive pathologies were classified as the NMDG. Parathyroid lesions were searched according to the surgery and pathology site. The lesion to background ratio (LBR), maximum diameter, volume, the mean CT Hounsfield unit values (CT_mean_) and location of each lesion were measured and observed. Regions of interest (ROIs) in the lesion and background (adjacent muscles) were manually delineated on SPECT/CT axial images slice by slice. The maximum of radioactivity counts was selected to represent MIBI uptakes of lesion and background, and the LBR was calculated [17, 18]. The length, height, width and CT_mean_ of the lesions were measured on CT images. The maximum of length, height, and width was the maximum diameter of the lesions. The volume was calculated by the equation for an ellipsoid model: volume = π/6 × (length × height × width) [19]. The location of eutopic lesions referred to four positions on the back of the thyroid glands: upper left, lower left, upper right and lower right. The parathyroid lesions in other locations were ectopic.

### Statistical analysis

Data were analyzed using Statistical Package for Social Sciences (SPSS version 20). Continuous variables were expressed as the mean ± standard deviation (SD). Categorical variables were expressed as numbers (percentages). Groups were compared with the Chi-square test, Mann–Whitney *U* test and Student’s *t* test, as appropriate. The sensitivity and 95% confidence interval (CI) were determined according to the pathological results. Differences in sensitivity between imaging methods were compared by using the McNemar test. Consistency of ^99m^Tc-MIBI SPECT/CT results of different observers were evaluated by the Kappa test. *P* values < 0.05 were considered statistically significant.

## Results

### Patients and lesions

A total of 46 patients underwent surgery, and 170 lesions were resected. Patient characteristics are summarized in Table 1. On the patient-based analysis, 78.3% of patients (36/46) had four lesions with pathological evidence. In the 36 patients, the proportion of all four lesions being detected by ^99m^Tc-MIBI SPECT/CT was 41.67%.

**Table 1:** Characteristics of the patients

Characteristics	All patients	MDG (n = 25)	NMDG (n = 21)
Sex			
Male	22 (47.8%)	14 (56.0%)	8 (38.1%)
Female	24 (52.2%)	11 (44.0%)	13 (61.9%)
Age (year)	46.46 ± 12.34	43.16 ± 11.89	48.19 ± 12.59
Years of CKD	8.00 ± 3.76	7.36 ± 3.49	8.76 ± 4.01
Years of dialysis	7.30 ± 3.84	6.40 ± 3.43	8.38 ± 4.11
Serum PTH (ng/ml)	2330.32 ± 1597.64	1950.04 ± 1064.08	2783.03 ± 1997.08
Serum alkaline phosphatase (U/L)	454.14 ± 500.74	494.67 ± 531.05	402.95 ± 468.79
Serum phosphorus (mmol/L)	1.97 ± 0.53	2.05 ± 0.34	1.87 ± 0.62
Serum calcium (mmol/L)	2.50 ± 0.25	2.43 ± 0.22	2.58 ± 0.27
Thyroid disease			
With	17 (37.0%)	10 (40.0%)	7 (33.3%)
Without	29 (63.0%)	15 (60.0%)	14 (66.7%)

*MDG* missed diagnosis group, *NMDG* non-missed diagnosis group, *CKD* chronic kidney disease, *PTH* parathyroid hormone

Of the 170 resected lesions, 160 lesions were proven parathyroid hyperplasia, 5 lesions were parathyroid adenoma, 3 lesions were thyroid tissue, 1 lesion was a lymph node, and 1 lesion was denatured fibrous adipose tissue. Of the 165 resected parathyroid lesions, no nodule with focal activity was found on SPECT/CT images corresponding to the surgical areas of 6 resected lesions, and two or more nodules with focal activity on SPECT/CT images corresponding to the surgical areas of 2 resected lesions. Finally, 157 identified lesions were included in the MDG and NMDG. There were 2 ectopic parathyroid lesions, all of which were parathyroid hyperplasias. One was located behind the left submandibular gland and the other was located in the right upper mediastinum.

### Diagnostic performance of different imaging methods

The diagnostic performance of different imaging methods based on the lesions is shown in Table 2. The sensitivity of ^99m^Tc-MIBI SPECT/CT were higher than those of ^99m^Tc-MIBI planar scintigraphy and US with significant differences (*P* < 0.05). The sensitivity of US was higher than that of ^99m^Tc-MIBI planar scintigraphy with significant differences (*P* = 0.005). The sensitivity of ^99m^Tc-MIBI SPECT/CT combined ^99m^Tc-MIBI planar scintigraphy was higher than ^99m^Tc-MIBI planar scintigraphy with significant difference (*P* < 0.001). The sensitivity of ^99m^Tc-MIBI SPECT/CT combined ^99m^Tc-MIBI planar scintigraphy was higher than ^99m^Tc-MIBI SPECT/CT with no significant difference (*P* = 0.250). The sensitivity of ^99m^Tc-MIBI SPECT/CT combined US was higher than ^99m^Tc-MIBI SPECT/CT or US alone with significant difference (*P* < 0.001).

**Table 2:** Diagnostic performance of different imaging methods

Imaging technology	Sensitivity (%) (95%CI)
^99m^Tc-MIBI SPECT/CT	70.3 (63.26–77.35)
^99m^Tc-MIBI planar scintigraphy	48.48 (40.78–56.19)
US	61.82 (54.33–69.31)
^99m^Tc-MIBI SPECT/CT and ^99m^Tc-MIBI planar scintigraphy	72.12 (65.21–79.03)
^99m^Tc-MIBI SPECT/CT and US	79.39 (73.16–85.63)

*US* ultrasonography, *CI* confidence interval

### Comparison between MDG and NMDG on ^99m^Tc-MIBI SPECT/CT images

On the lesion-based analysis, as shown in Table 3, 157 parathyroid lesions were divided into an MDG (n = 41) and NMDG (n = 116). The LBR, maximum diameter and volume of lesions in MDG was smaller than those in NMDG with significant difference (*P* < 0.001). There was no significant difference in CT_mean_ and location between the two groups (*P* > 0.05). Different observers had moderate consistency in ^99m^Tc-MIBI SPECT/CT results by the Kappa test (*κ* = 0.560).

**Table 3:** Characteristics of the lesions in the MDG and NMDG on 99mTc-MIBI SPECT/CT images

Characteristics	MDG (n = 41)	NMDG (n = 116)	*P*
LBR	3.42 ± 1.28	6.75 ± 5.08	< 0.001*
Maximum Diameter (mm)	9.32 ± 2.69	15.03 ± 4.94	< 0.001*
Volume (mm^3^)	208.51 ± 163.22	863.85 ± 1216.0	< 0.001*
CT_mean_ (HUs)	51.77 ± 17.49	57.12 ± 15.38	0.067
Location			0.119
Upper left	13 (31.7%)	22 (19.0%)	
Upper right	13 (31.7%)	35 (30.2%)	
Lower left	7 (17.1%)	24 (20.7%)	
Lower right	8 (19.5%)	33 (28.4%)	
Ectopic	0 (0.0%)	2 (1.7%)	

*MDG* missed diagnosis group, *NMDG* non-missed diagnosis group, *LBR* the lesion to background ratio, *HUs* Hounsfield units

^*^P < 0.05, MDG versus NMDG

## Discussion

^99m^Tc-MIBI SPECT/CT, as a classical nuclear medical imaging technique, is often used for preoperative localization in patients with SHPT [14, 15]. In our study, as shown in Table 2, the sensitivity of ^99m^Tc-MIBI SPECT/CT was higher than that of ^99m^Tc-MIBI planar scintigraphy and US in SHPT based on lesions. Previous studies [12, 13] found that the sensitivity of ^99m^Tc-MIBI planar scintigraphy was lower than that of US or ^99m^Tc-MIBI SPECT/CT in SHPT. Our results were consistent with previous studies. ^99m^Tc-MIBI SPECT/CT had higher sensitivity than planar scintigraphy, which may be due to its ability to provide anatomical location and its superior resolution [22]. However, it is still controversial whether the sensitivity of ^99m^Tc-MIBI SPECT/CT is higher than that of US in SHPT [11, 21]. Our result was consistent with the study of Li et al. [11].

In our study, ^99m^Tc-MIBI SPECT/CT combined US had higher sensitivity than ^99m^Tc-MIBI SPECT/CT or US alone with significant difference. Li et al. [21] found that ^99m^Tc-MIBI SPECT/CT combined with US exhibit higher sensitivity than ^99m^Tc-MIBI SPECT/CT or US alone in SHPT. Our results were consistent with these results. In our study, ^99m^Tc-MIBI SPECT/CT combined planar scintigraphy had higher sensitivity than planar scintigraphy with significant difference. This result was easily understood due to the higher sensitivity of SPECT/CT compared to planar scintigraphy. In our study, ^99m^Tc-MIBI SPECT/CT combined planar scintigraphy had higher sensitivity than ^99m^Tc-MIBI SPECT/CT with no significant difference. Some parathyroid hyperplasia may rapidly wash out ^99m^Tc-MIBI [23], so it may lead to negative SPECT results. However, thyroid nodules may lead to positive planar imaging results. Therefore, this result was due to the limitations of SPECT/CT acquisition mode and planar imaging which can not provide anatomical location.

Although ^99m^Tc-MIBI SPECT/CT exhibited the highest sensitivity of 70.3% among the three methods, 29.7% of the parathyroid lesions were missed with ^99m^Tc-MIBI SPECT/CT. In addition, On the patient-based analysis, the majority of patients (78.3%) had multiglandular disease with four lesions, but the proportion of all four lesions being detected by ^99m^Tc-MIBI SPECT/CT was only 41.67%. These results indicate that ^99m^Tc-MIBI SPECT/CT missed some lesions in patients with SHPT, especially in those with multiglandular disease, which was consistent with previous study [11]. In our study, different observers showed moderate interobserver reproducibility, which meant that there was no notable correlation between results of ^99m^Tc-MIBI SPECT/CT and the diagnostic level of physicians.

On the lesion-based analysis, of all resected parathyroid lesions, 6 missed lesions were not found on SPECT/CT images maybe due to the limitation of spatial resolution and 2 missed lesions were difficult to distinguish from the surrounding soft tissues in the same location. Finally, 157 identified lesions were included in the in the analysis of missed diagnoses. We included the LBR, maximum diameter, volume, CT_mean_ and location of the 157 lesions. Previous studies have suggested that parathyroid lesions with lower LBRs or smaller parathyroid lesions tend to be missed [11, 13, 17, 18]. In addition, the adjacent tissues varied in different locations, and we suspected that the adjacent tissues with similar parathyroid density may lead to missed diagnoses. Therefore, the LBR, maximum diameter, volume, CT_mean_ and location were compared between the MDG and NMDG.

**Fig.1:**
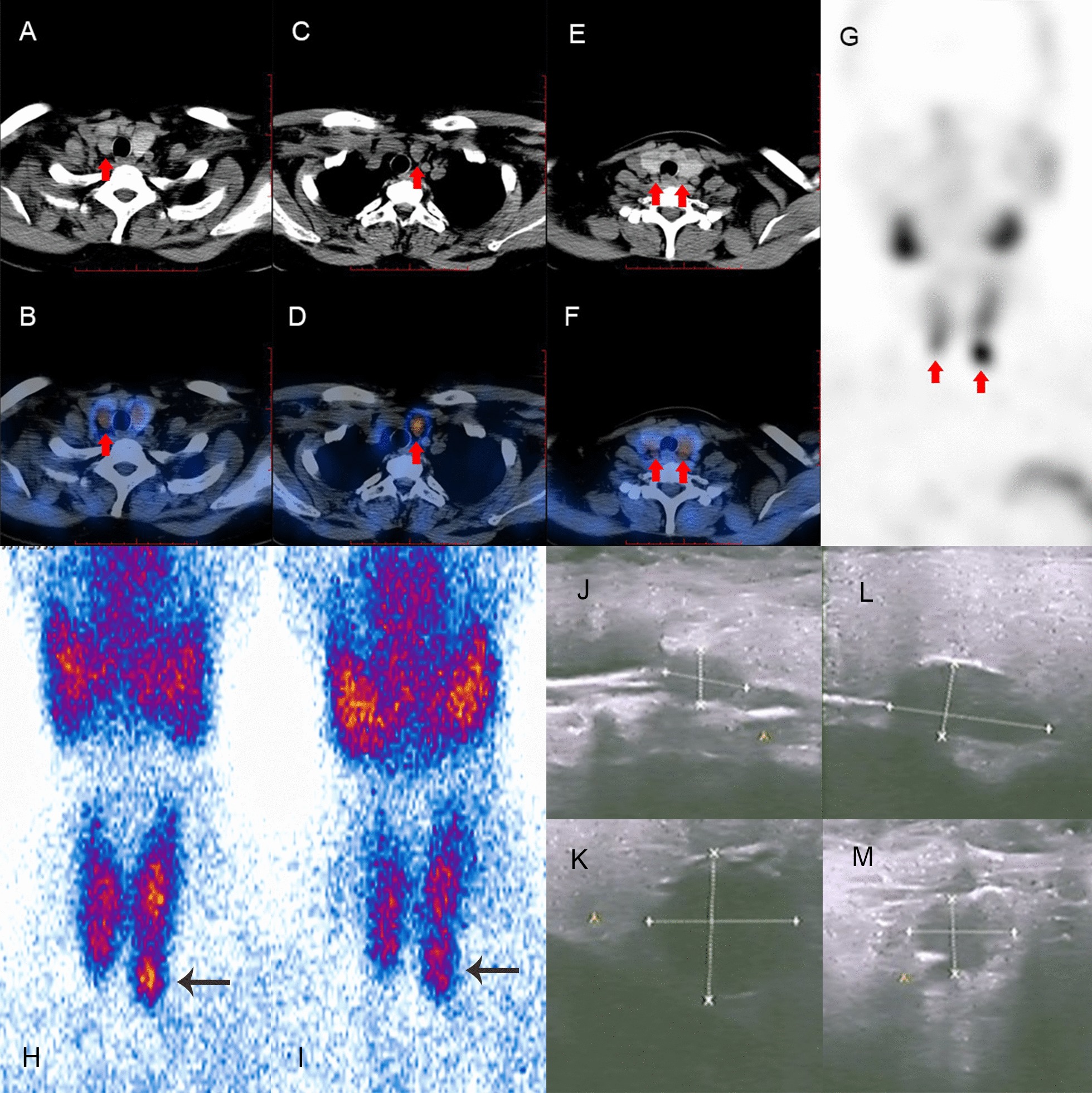
A middle-aged patient suffered from SHPT. The axial CT (**a** and **c**) and SPECT/CT (**b** and **d**) images showed two large soft-tissue masses with marked MIBI uptake in the back of the inferior poles of bilateral thyroid lobes (arrows). The axial CT (**e**) and SPECT/CT (**f**) images showed two small soft-tissue masses with slight MIBI uptake in the back of the upper poles of bilateral thyroid lobes (arrows). The maximum density projection image (**g**) only showed two foci in the inferior pole of bilateral thyroid lobes (arrows). Four lesions in the back of the thyroid gland were pathologically confirmed to be parathyroid hyperplasia. In the initial diagnosis, the lower left and right lesions were diagnosed correctly, but the upper left and right lesions were missed. The early and late planar images (**h** and **i**) showed MIBI uptake in the inferior pole of left thyroid lobe (arrows). US images (**j–m**) showed 4 enlarged parathyroid glands in the back of the upper and inferior poles of bilateral thyroid lobes.

The results showed that the LBR, maximum diameter and volume of lesions were significantly different between the two groups. As shown in Fig. 1, poor visualization of these lesions with lower LBR and smaller lesions led to missed diagnoses, which was easy to understand. However, the CT_mean_ and location were not significantly different between the groups. Marmin et al. [20] believed that the density of parathyroid adenomas was significantly lower than that of thyroid parenchyma, which indicated that the probability of missed diagnoses due to the similar density between the thyroid and parathyroid lesions was low. Nichols et al. [16] found that the sensitivity of ^99m^Tc-MIBI SPECT/CT in diagnosing primary hyperparathyroidism was statistically similar for all locations. The results suggested that the location had little effect on missed diagnoses.

As Table 3 showed, the average maximum diameter of NMDG was 15.03 ± 4.94 mm. Li et al. [11] reported that 8.05 mm was the optimal cut-off value in lesions diameter for predicting ^99m^Tc-MIBI SPECT/CT positive in SHPT. Zhou et al. [24] reported that the average maximum diameter of ^99m^Tc-MIBI SPECT/CT positive lesions was 19.6 ± 9.5 mm in hyperparathyroidism. NMDG refered to true positive lesions, while MIBI positive included ture positive and false positive lesions. The different results between our study and previous studies may be due to the different subjects. In our study, the average volume of NMDG was 863.85 ± 1216.0 mm^3^, and the average LBR of NMDG was 6.75 ± 5.08. This was the first one that compared the volume and LBR of MGD and NMGD on ^99m^Tc-MIBI SPECT/CT.

Zhang et al. [25] found that the sensitivity of early ^99m^Tc-MIBI SPECT/CT was higher than delayed ^99m^Tc-MIBI SPECT/CT in patients with SHPT. Krakauer et al. [26] reported that dual-isotope subtraction scintigraphy had higher diagnostic accuracy than dual-phase scintigraphy in patients with primary hyperthyroidism. Therefore, compared with delayed ^99m^Tc-MIBI SPECT/CT, early ^99m^Tc-MIBI SPECT/CT may increase the sensitivity in patients with SHPT. The diagnostic performance of dual-isotope subtraction scintigraphy in patients with SHPT remains to researched.

There were still some limitations in our study. First, this was a retrospective single-center study, which may result in some selection bias. Second, SPECT/CT was performed at 120 min after injection of ^99m^Tc-MIBI in our study. As parathyroid hyperplasia, which is common in SHPT, may rapidly washed out ^99m^Tc-MIBI [23], so it may lead to some parathyroid lesions missed. Thirdly, we did not analyze the false positive results of ^99m^Tc-MIBI SPECT/CT based on lesions, which often occur with thyroid nodules. Finally, our subjects were all patients with hyperparathyroidism secondary to CKD with relatively limited samples. Multicenter studies with larger samples of hyperparathyroidism secondary to other diseases need to be carried out in the future.

## Conclusions

In conclusion, ^99m^Tc-MIBI SPECT/CT showed a higher sensitivity than ^99m^Tc-MIBI planar scintigraphy and US in patients with SHPT, and the combination of ^99m^Tc-MIBI SPECT/CT and US appeared to futher increase the sensitivity. On ^99m^Tc-MIBI SPECT/CT images, it is necessary to pay attention to those parathyroid lesions with lower MIBI uptake and smaller parathyroid lesions to avoid missed diagnoses.

## Data Availability

The datasets generated and/or analysed during the current study are available from the corresponding author on reasonable request.

## Abbreviations

SHPT: Secondary hyperparathyroidism
CKD: Chronic kidney disease
PTH: Parathyroid hormone
US: Ultrasonography
MDG: Missed diagnosis group
NMDG: Non-missed diagnosis group
LBR: The lesion to background ratio
CTmean: The mean CT Hounsfield unit values
ROIs: Regions of interest
SD: Standard deviation
CI: Confidence interval
